# Heat-killed salmonella typhimurium (HKST) protects mice against radiation in TLR4-dependent manner

**DOI:** 10.18632/oncotarget.17859

**Published:** 2017-05-15

**Authors:** Yang Xu, Yuanyuan Chen, Hu Liu, Xiao Lei, Jiaming Guo, Kun Cao, Cong Liu, Bailong Li, Jianming Cai, Jintao Ju, Fu Gao, Yanyong Yang

**Affiliations:** ^1^ Department of Radiation Medicine, Faculty of Naval Medicine, Second Military Medical University, Shanghai 200433, P.R. China; ^2^ Faculty of Naval Medicine, Second Military Medical University, 200433, Shanghai, P.R. China

**Keywords:** HKST, radioprotection, TLR, radiosensitive tissues, DNA damage

## Abstract

It is urgently required to develop novel safe and effective radioprotectors to alleviate radiation damages. Recently, several toll like receptors (TLRs), including TLR2, TLR4, TLR5, TLR9, have been proved to exert protective effects against ionizing radiation. Due to different tissue-distribution and distinct functions of TLRs, we hypothesized that co-activation of multiple TLRs simultaneously may produce extensive and stronger radioprotective effects. In this study, we found the co-agonist of TLR2, TLR4 and TLR5, heat-killed salmonella typhimurium (HKST) significantly inhibited radiation-induced cell apoptosis, increased cell survival and alleviated DNA damage. HKST also prolonged animal survival and protected radiosensitive tissues against radiation damages, such as bone marrow, spleen and testis. Decrease of CD4+ and CD8+ cells were also reversed by HKST treatment. By using TLR2 and TLR4 knockout mice, we found that most of radioprotective effects of HKST were abrogated in TLR4 knock out mice. And HKST failed to inhibited cell apoptosis in TLR5 knock down cells. In conclusion, we demonstrated that HKST effectively protected cells and radiosensitive tissues against radiation injury in a TLR4 biased mechanism, suggesting HKST as a potential radioprotector with low toxicity.

## INTRODUCTION

Disasters from nuclear reactor meltdown, dirty bomb or nuclear bomb explosion emit lots of ionizing radiation, exposure to which causes acute and chronic radiation sickness. In cancer radiotherapy, normal tissue toxicity still remains a major dose-limiting factor. In general, it is believed that the critical target of ionizing radiation (IR) is DNA [1, 2]. The radiolysis of water by IR produces free radicals, which induces irreparable DNA damage [3]. Unrepaired or error-repaired DNA damage can initiate a chain of events leading to cellular damage or death [4]. Previously, most radioprotective drugs mainly focused on synthesized or natural compounds with free radical scavenger/antioxidant capacity [5].

Recent years, damage-associated molecular pattern molecules in particular toll-like receptors (TLRs) provide novel insights on radioprotection. Several studies have proved that TLR5 ligand exerted radioprotective capacity in mouse and monkey models [6]. Our group reported that TLR2, TLR4 and TLR9 also play critical roles in radio-resistance [7–9]. However, different TLRs recognize specific component of pathogens, and initiate distinct downstream signaling pathways. The distribution and abundance of different TLRs also varies in different organs. Thus, we hypothesized that co-activation of multiple TLRs may exert stronger and more extensive radioprotective effects than single TLR agonist. For example, it has also been reported that co-agonist of TLR2 and TLR4, Escherichia coli O111:B4 LPS was stronger than any single TLR2 or TLR4 agonist or the combination of both in terms of immune stimulation [10].

Previous studies have confirmed that heat-killed salmonella typhimurium (HKST) is an effective TLR2, TLR4 and TLR5 co-agonist, which effectively activates the downstream signaling pathways [11]. TLR2 and TLR4 recognize cell wall components from HKST, such as peptidoglycan (PGN) and lipopolysaccharide (LPS). TLR5 recognizes extracellular flagellin, resulting in NF-κB activation. In current study, we are aimed to test the potential protective effects of HKST on cells and radiosensitive tissues in mice against γ-irradiation. To distinguish the underlying mechanism, we investigated the changes of NF-kB and MAPK signaling pathways. And TLR2, TLR4 and TLR5 knock down cells were also used to determine their relative contributions.

## RESULTS

### HKST protected cells against radiation-induced cell death and DNA damage

**Figure 1:**
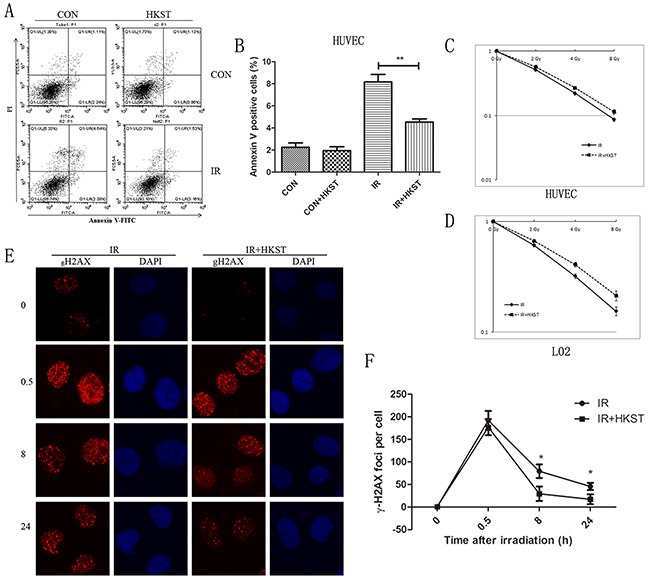
HKST protected cells against radiation-induced cell death and DNA damage HUVEC cells were pretreated with HKST (107/mL) for 12h and exposure to IR. Cell apoptosis were determined at 24h post irradiation by flow cytometry **(A)**. Annexin V positive cells were quantified in different groups **(B)**. HUVEC cells **(C)** and L02 cells **(D)** were exposed to 0, 2, 4, 8Gy irradiation and colony formation assay were used to measure cell survival. A γ-H2AX foci assay was used to determine DNA damage in HUVEC cells at 0, 0.5, 8, 24h after exposure to 3Gy irradiation **(E)**. The numbers of γ-H2AX foci per cell in different groups were counted **(F)**. (n=6) *P<0.05, **P<0.01 Vs IR groups.

To investigate whether HKST has *in vitro* radioprotective capacity, we measured cell apoptosis in human HUVEC and RAW264.7 cells. At 24h after irradiation, cell apoptosis rate obviously increased, while in cells treated with HKST, the apoptosis significantly inhibited (Figure 1A, 1B). We also found that HKST significantly increased cell survival after IR in HUVEC and L02 cells (Figure 1C, 1D). It is well known that DNA is a main target of IR and efficacy of DNA damage repair determines cell fate. So we evaluate the influence on DNA repair of HKST thgough γ-H2AX foci kinetics [12]. Our results showed that HKST reduced the number of γH2AX foci per cell at 8h and 24h post-irradiation (Figure 1E, 1F), indicating a role of HKST in promoting DNA damage repair.

### HKST activated nuclear factor kappa B (NF-kB) and MAPK signaling pathway

**Figure 2:**
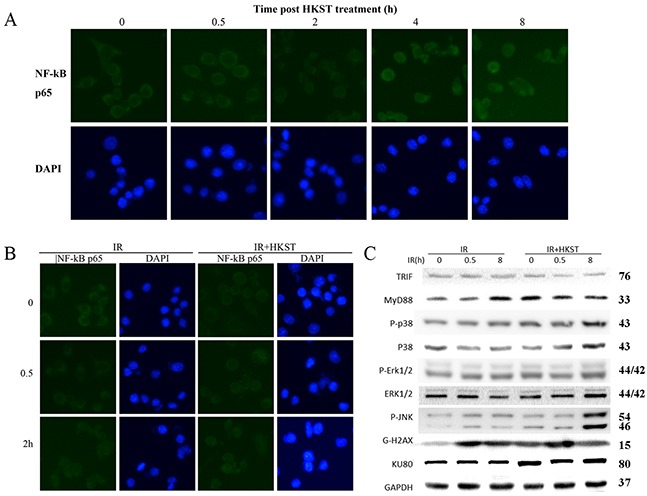
HKST activated NF-kB and MAPK signaling pathway At 0, 0.5, 2, 4, 8h post HKST treatment, RAW264.7 cells were stained with a NF-kB p65 antibody and related secondary antibodies by using an immunofluenrence staining assay **(A)**. Location of p65 was also stained in IR group and IR+HKST group **(B)**. The effects of HKST on TRIF, MyD88 and MAPK signaling pathway at 0, 0.5, 8h after irradiation were measured by a western blot assay **(C)**. (n=5).

Several studies have demonstrated that activation NF-kB accounts for the radioprotective capacity of TLRs. And through a NF-kB p65 immunofluenrence staining assay, we observed significant translocations of NF-kB p65 at 4h and 8h after HKST treatment, indicating HKST effectively activated NF-kB (Figure 2A). The activation of NF-kB was much earlier in HKST treated group than that in IR group (Figure 2B). In addition, we found that HKST elevated the level of MyD88, while showed little influence on TRIF (Figure 2C). Our data also showed that HKST enhanced the phosphorylation of JNK and p38 at 8h post irradiation (Figure 2C). These data provided possible mechanisms for its radioprotective effects.

### HKST mitigated destructions of bone marrow and increased nucleated cells following irradiation

**Figure 3:**
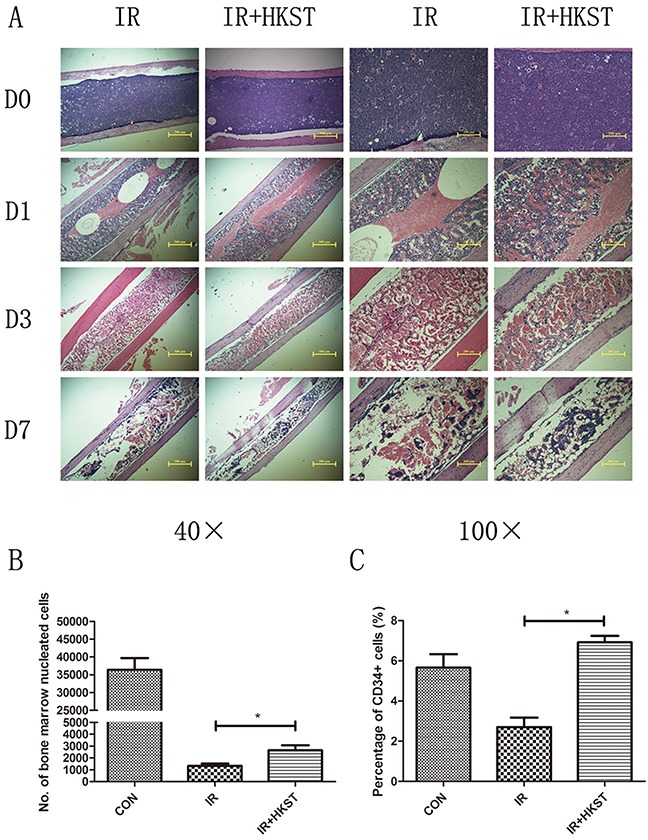
HKST mitigated destructions of bone marrow and increased nucleated cells following irradiation Mice were pretreated with HKST or PBS for 12h and then exposed to 6Gy γ-irradiation. On day1, day3 day7 post-irradiation, femurs were isolated and subjected to tissue sectioning and HE staining **(A)**. On the 3^rd^ day post-irradiation, bone marrow nucleated cells were counted by flow cytometry **(B)** and CD34+ HSC were also analyzed by a flow method **(C)**. (n=8). *P<0.05 Vs IR groups.

It has been well established that IR causes a drastic deficit in the hematopoietic system, which may cause dysfunction to bone marrow cells populations. To determine whether HKST could alleviate radiation damages on bone marrow, we analyzed structure of bone marrow and nucleated cells counts on the 3^rd^ day post 6Gy irradiation. We found that in HKST treated group, the damage of IR on bone marrow structure was mitigated, and the decrease of nucleated cells was also significantly inhibited (Figure 3A, 3B). In addition, we found that HKST treatment increased the number of CD34+ HSC (Figure 3C).

### HKST alleviated radiation damages on spleen and immune dysfunction

**Figure 4:**
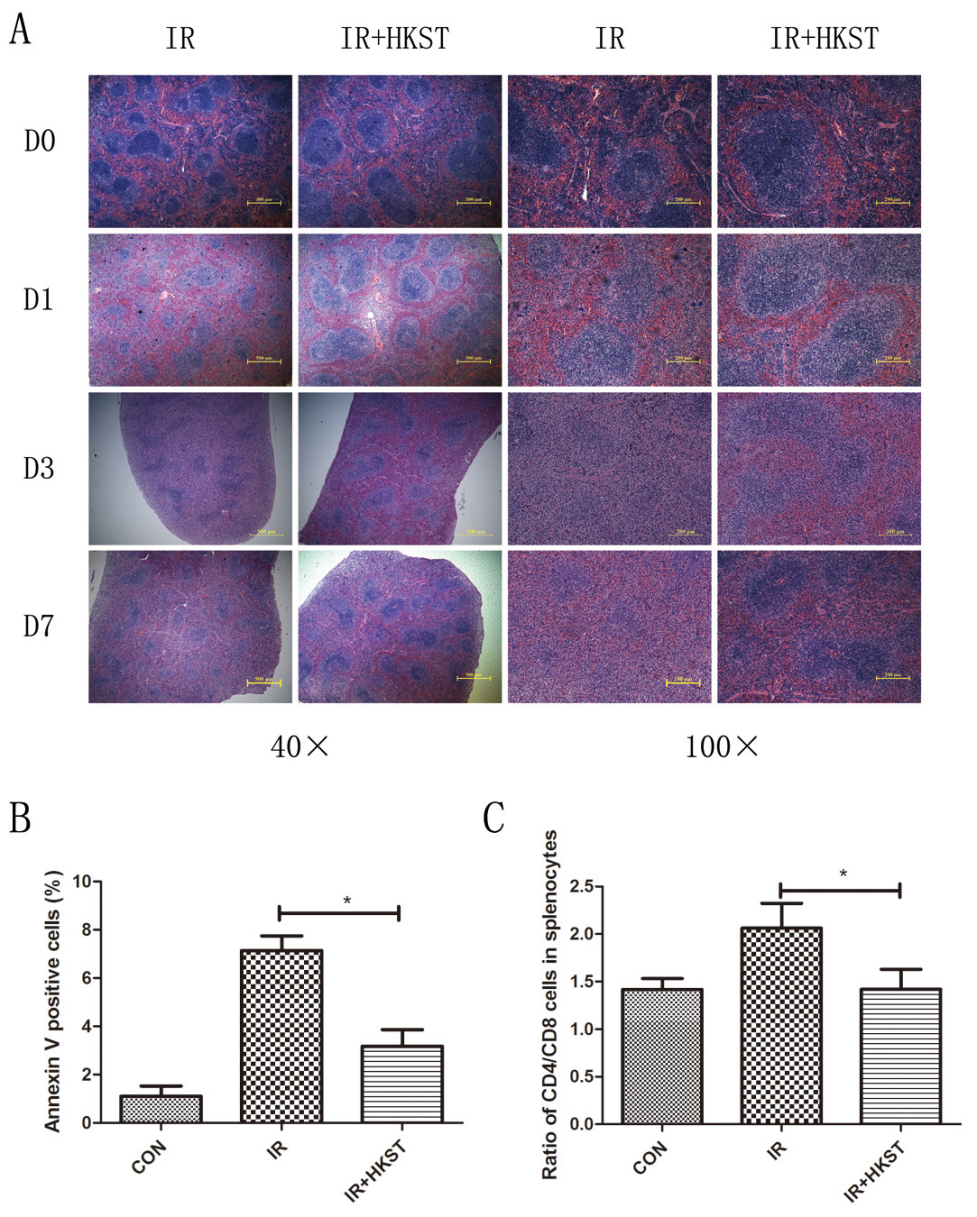
HKST alleviated radiation damages on spleen and immune dysfunction Spleens were isolated from mice with and without HKST treatment on day1, day3 day7 post-irradiation and subjected to HE staining **(A)**. Apoptosis of splenocytes was measured by a flow cytometry method at 12h after irradiation and Annexin V positive cells were analyzed **(B)**. Ratio of CD4+ and CD8+ splenocytes was also analyzed in different groups. (n=8). *P<0.05 Vs IR groups.

Immunocytes are quite sensitive to IR, which could also result in immune dysfunction. We examined the damages of spleen structure and function to determine whether HKST protect immunocytes in response to IR. As shown in Figure 4A, size of white pulps reduced obviously after IR compared to the Control groups, while in HKST treated group, the structure of spleen was protected. The apoptotic spleen cells rate was significantly inhibited by HKST (Figure 4B). These results suggested that HKST could protect splenocytes from radiation-induced injury through inhibiting apoptosis. To further explore the effects of HKST on immune imbalance induced by IR, the percentage of CD4+ and CD8+ T cells in splenocytes were measured. As shown in Figure 4C, the ratio of CD4+/CD8+ cells in radiation group were higher than that in control group, but the ratio reduced significantly in IR+HKST group.

### HKST protected testis damage induced by irradiation and increased animal survival

**Figure 5:**
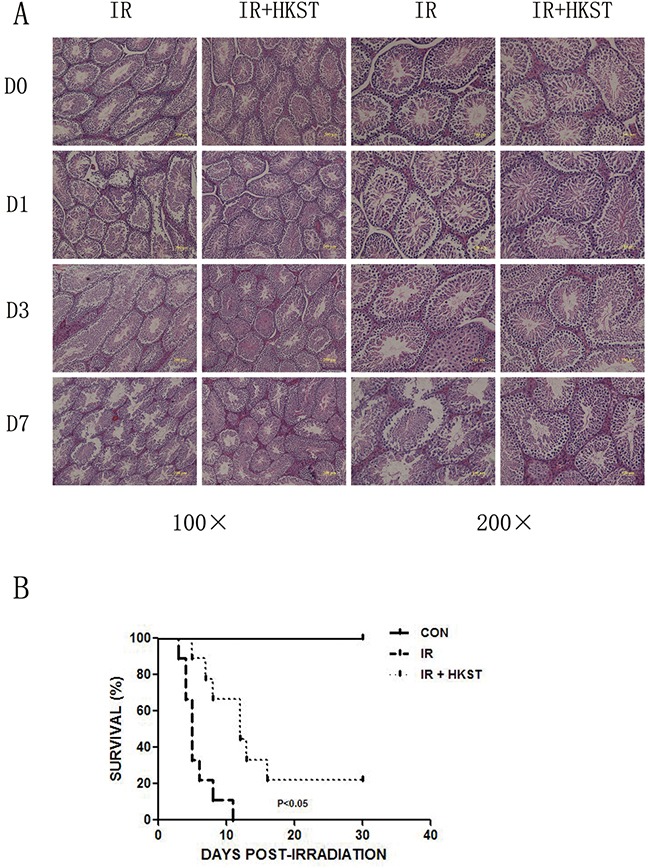
HKST protected testis damage induced by irradiation and increased animal survival Testis were isolated from mice with and without HKST treatment on day1, day3 day7 post-irradiation and subjected to HE staining **(A)**. (n=8) After 7Gy irradiation, animal survival was recorded up to 30 days **(B)**. (n=15).

Ionizing radiation has long been regarded as an iatrogenic male reproductive toxin, especially rapidly proliferating spermatogenic cells. Even low doses of radiation can cause considerable functional impairment of the testis [13]. We found that pretreatment with HKST protected testis from destruction, and reduced the condensation of nuclear chromatin (Figure 5A). We also conducted a survival assay and found that in HKST treated group, survival of mice increased significantly in response to 7Gy γ-irradiation (Figure 5B).

### HKST inhibited inflammatory cytokines induced by irradiation

**Figure 6:**
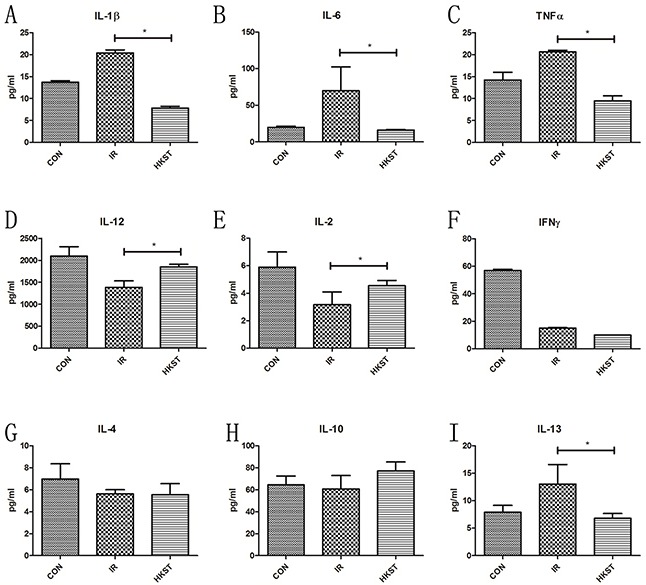
HKST modulated inflammatoty cytokines induced by irradiation At 24h post-irradiation, cytokines in blood serum of mice from different groups were measured by an ELISA assay **(A-I)**. (n=8) *P<0.05, **P<0.01 Vs IR groups. (n=8).

Inflammatory cytokines play important roles in the progression of radiation sickness and hematopoietic regulation [14]. As shown in Figure 6 A-C, IL-1β, IL-6 and TNF-α in the serum was elevated in IR group, while in IR+HKST group the levels were significantly inhibited. Increase of IL-12, IL-2 and IFN-γ was observed in HKST pretreated mice as compared with mice in IR group. However, increase in IL-13 expression was observed in irradiated mice, which was found to be subsidized by HKST treatment. These data indicates that HKST pretreatment reversed Th1/Th2 imbalance and suppressed radiation-induced inflammation.

### Radioprotective effects of HKST were mostly abrogated in TLR4−/− mice

**Figure 7:**
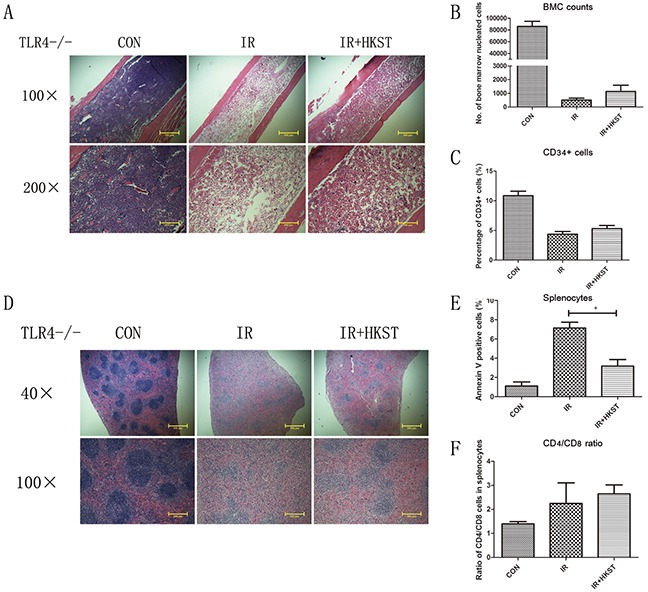
Radioprotective effects of HKST were mostly abrogated in TLR4−/− mice Femurs **(A)** and spleens **(D)** from irradiated TLR4−/− mice with and without HKST treatment (10^8^/mouse) were isolated and stained with a HE method. Bone marrow nucleated cells per femur **(B)** and the percentage of CD34+ HSC **(C)** were analyzed by flow cytometry. For splenocytes, cell apoptosis **(E)** and CD4+/CD8+ cells ratio **(F)** was measured by flow cytometry at 24h after irradiation. (n=8). *P<0.05 Vs IR groups.

Our previous study had shown that mice deficient in TLR4 were very susceptible to IR-induced mortality. HKST was proved efficiently activating TLR4 and TLR2, and we used TLR4−/− mice to determine the role of TLR4 in the radioprotection of HKST. We found that HKST showed little protective effects on bone marrow structure in HE sections, as well as the number of nucleated cells and CD34+ cells in TLR4−/− mice, compared with that in wild type mice (Figure 7A-7C). However, we found that the size of white pulps was slightly protected by HKST (Figure 7D). But no significant difference was found in the apoptosis of splenocytes between IR group and IR+HKST group, either the CD4+ and CD8+ cells ratio (Figure 7E, 7F). These data indicated that TLR4 played a key role in mediating the radioprotective effects of HKST.

### The radioprotective effects of HKST were partially dependent on TLR2

**Figure 8:**
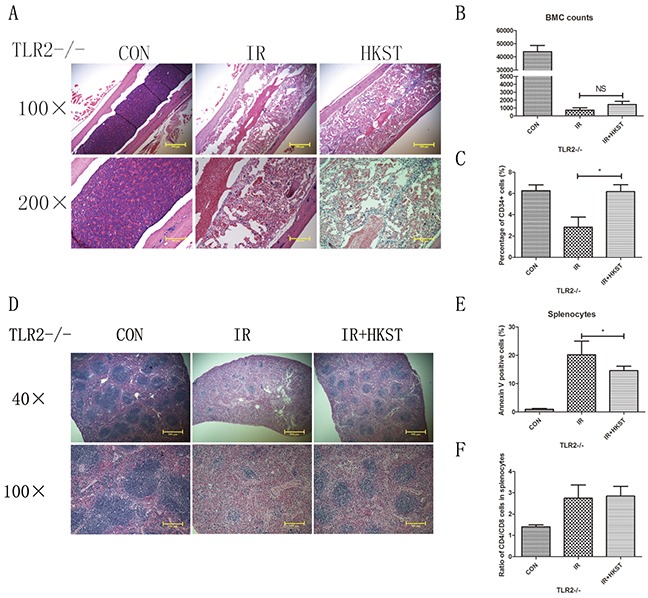
Radioprotective effects of HKST were partially dependent on TLR2 In TLR2−/− mice, femurs **(A)** and spleens **(D)** were isolated from different groups on the 3^rd^ day post-irradiation, and subjected to HE staining. Flow cytometry were used to analyze the number of nucleated cells in bone marrow **(B)** and the percentage of CD34+ cells **(C)**. Splenocyte apoptosis **(E)** and the CD4+/CD8+ cells ratio **(F)** in spleen were also measured at 24h post-irradiation. (n=8). *P<0.05 Vs IR groups.

Our group had revealed that TLR2 contributed to cellular resistance to IR. So we investigated the possible role of TLR2 in mediating the radioprotective effects of HKST. In TLR2−/− mice, we found that HKST significantly protected splenocytes from radiation-induced apoptosis (Figure 8E), as well as the structural changes of spleen sections (Figure 8D). HKST also effectively inhibited the loss of CD34+ cells in response to IR (Figure 8C). But no difference in the ratio of CD4+ and CD8+ cells, or nucleated bone marrow cells was found (Figure 8A, 8B, 8F).

### Radioprotective effects of HKST were dependent on TLR4 and TLR5 *in vitro*

**Figure 9:**
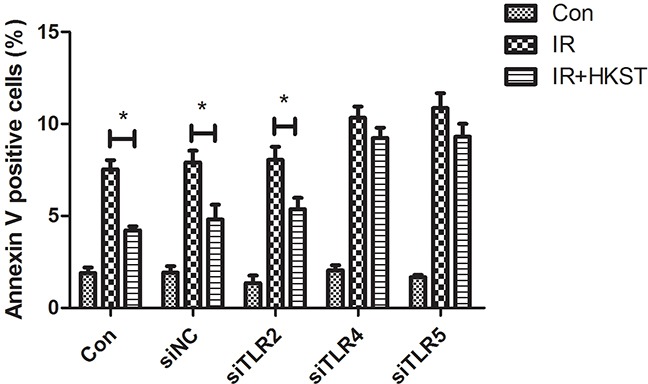
HKST inhibited radiation-induced apoptosis in TLR2 knock down cells, but not in TLR4 and TLR5 knock down cells To determine the underlying mechanism of HKST, we used TLR2 siRNA, TLR4 siRNA or TLR5 siRNA to knock down TLR2, 4, 5 in HUVEC cells. At 72h after siRNA transfection, cells were irradiated at the dose of 8Gy after 12h HKST treatment. 24h later, cell apoptosis was determined with flow cytometry and Annexin V positive cells were analyzed. (n=6) *P<0.05 Vs IR groups.

We used TLR2, TLR4 and TLR5 siRNA to knock down these TLRs separately in HUVEC cells. Our data showed that HKST significantly inhibited cell apoptosis in siNC and siTLR2 transfected cells after irradiation. But no significant difference was found between IR and IR+HKST group in TLR4 or TLR5 knock down cells (Figure 9). These data suggests that TLR4 and TLR5 are potential targets of HKST in radioprotection.

## DISCUSSION

Exposure to IR induces various pathophysiological changes, including direct DNA damages and production of free radicals, which contribute to radiation injury in multiple tissues [15]. Toll-like receptor (TLR) is a class of innate immune sensors which can direct adaptive immune responses. It has been reported that ligand of TLR2, TLR4, TLR5, or TLR9 exhibited radioprotective effects *in vitro* and *in vivo*. Previous studies have shown that heat killed salmonella typhimurium (HKST) is an effective TLR2/4/5 co-agonist, which simultaneously activates TLR2/4/5 and their downstream signaling pathways [16]. In this study, we demonstrated that HKST effectively protected cultured cells and mice against radiation injuries *in vitro* and *in vivo*, mainly in a TLR4 biased mechanism.

As IR can disrupt the structure and function of DNA and proteins, which impairs the metabolic functions and lead to cell apoptosis, senescence and necrosis. In this study, our data showed that HKST increased cell survival and inhibited cell apoptosis after exposure to irradiation. DNA is the most critical target for IR, exposure to which results in DNA double-strand breaks (DSB) and cell apoptosis. The number of γH2AX foci was used as a marker of DSBs [17], and in present study, we analyzed γH2AX kinetics in HUVEC cells. The results indicated that HKST alleviated DNA damage in response to IR. As HKST mainly activates TLRs, and the relationship between TLR signaling pathway and DNA repair needs to be clarified.

The hematopoietic system and immune system are very sensitive to IR. IR could result in progressing loss of bone marrow nucleated cells as well as immunosuppression. Our present study proved that HKST treatment increased the number of bone marrow nucleated cells as well as CD34+ hematopoietic stem cells (HSC), and inhibited cell apoptosis in splenocytes. We also found that radiation induced imbalance of CD4 and CD8 cells, while was reversed by HKST treatment. We also used an ELISA assay to measure the concentrations of serum cytokines. The results showed that HKST played anti-inflammatory roles by suppressing IL-6 and TNF-α. These findings suggested that HKST treatment protected hematopoiesis damages and immune dysfunctions caused by IR.

It has been reported that TLRs mainly trigger myeloid differentiation factor 88 (MyD88) and TRIF dependent signaling pathway [18]. After TLRs binding with their ligands, their downstream mediators phosphorylates IKK-β and mitogen-activating protein kinases (MAPKs) including ERK1/2, c-jun terminated kinase (JNK)1/2 and p38, leading to activation of nuclear factor kappa B (NF-κB) and activating protein (AP)-1 [19]. In our present study, we found that HKST treatment obviously increased the expression of MyD88, and the phosphorylation of p38 and JNK. We also observed HKST treatment induced translocation of NF-kB p65 subunit, which accounts for radioprotective effects of TLRs.

As HKST possess both TLR4 and TLR2 activities, however, contribution of TLR2 or TLR4 to the radioprotective effects of HKST is still unknown. We used TLR2 and TLR4 knockout mice to further explore the underlying mechanism. Our data suggested a TLR4 biased mechanism for the radioprotective effects of HKST, as no protective effect was observed in TLR4−/− mice. In addition, the radioprotective effects of HKST were partially abrogated in TLR2 knockout mice, suggesting TLR2 also contributed to radioprotection of HKST. But the underlying mechanism of TLR signaling pathway in radioprotective effects of HKST needs to be further examination. It was proved that HKST activates TLR5 at high concentration. Next, we used TLR2 siRNA, TLR4 siRNA and TLR5 siRNA to knock down these TLRs *in vitro*. We found that HKST significantly protected TLR2 knock down cells from radiation, while no difference was observed in TLR4 and TLR5 knock down cells, suggesting that HKST mainly targets TLR4 and TLR5 in radioprotection.

Safety is another requirement for an appropriate radioprotector. Several studies have confirmed attenuated Salmonella can be used as a therapeutic anti-tumor vaccine. As a good carrier with antigens and natural mucosal immune adjuvant, attenuated ST has wide applications in the field of vaccination. It was a relatively safe vaccine inducing Th1 immune responses to malignancies and microbes [20]. Our results also indicate that HKST exhibits protective effects on radiation-induced injury with low toxicity, suggesting great potentials in clinical uses for radioprotection.

In conclusion, our findings demonstrated that TLR2/4/5 co-agonist HKST protected against radiation-induced damages. We showed that HKST may protect cells and radiosensitive tissues against irradiation depend on TLR4 and TLR5 signaling pathway. HKST can also exert anti-inflammatory through suppressing TNF-α and IL-6, with the increase of Th1-related cytokines. This suggests that HKST might be a novel safe and effective radioprotector.

## MATERIALS AND METHODS

### Cells and treatments

Human umbilical vein endothelial cells (HUVEC) and human liver L02 cells (American Type Culture Collection, Manassas, VA, USA) were maintained in Dulbecco’s modified eagle’s medium (DMEM) (PAA Laboratories) with 10% fetal bovine serum (PAA Laboratories) and 1% penicillin-streptomycin-glutamine (Hyclone, Logan, USA) at 37°C in a 5% CO_2_ humidified chamber. Murine macrophage cells (RAW 264.7) were maintained in the RMPI1640 medium in the same condition. After HKST (10^7^/mL) (Invivogen, US) treatment for 12h, cells were exposed γ-irradiation at the dose rate of 1Gy/min. For apoptosis assay and western blot assay, cells were exposed to 8Gy γ-irradiation. Survival curve was obtained by using HUVEC and L02 cells treated with 0, 2, 4, 8Gy γ-irradiation. For γH2AX foci, HUVEC cells were exposed to 3Gy of irradiation and subjected to Immunofluorescence analysis.

### Mice and treatments

All the experiments were approved by the Second Military Medical University, China in accordance with the Guide for Care and Use of Laboratory Animals published by the US NIH (publication No. 96-01). Male wild-type C57BL/6 mice (6-8 weeks old) were obtained from China Academy of Science (Shanghai, China). Toll like receptor 4 knock out (TLR4−/−) and TLR2−/− mice from the same background were purchased from Model Animal Research Center of Nanjing University. Mice were housed in individual cages in a temperature-controlled room with a 12h light/dark cycle. Food and water were provided ad libitum. Animals were divided into four groups as follows: the Control group, HKST group, IR group and IR+HKST group were irradiated. HKST dissolved in PBS (10^8^/mice) was delivered through intragastric administration at 12h before irradiation. The mice received 6Gy or 7Gy total-body irradiation in a holder designed to immobilize unanaesthetized mice such that the abdomens were exposed to the beam. The environment light intensity and temperature in irradiation center are kept the same as those in the animal house. At different time post-irradiation, mice were killed by cervical dislocation. For each time point, spleens, femurs and testis tissues were isolated from at least 10 mice. We used spleen, femur and testis for sectioning and HE staining. Bone marrow cells and splenocytes isolated from mice at the same time were used for flow cytometry analysis.

### Irradiation

^60^Co-γ rays in Irradiation Center (Faculty of Naval Medicine, Second Military Medical University, China) were used for the radiation purpose. Mice and cells were exposed to different doses of radiation, depending upon the requirement of the present study.

#### Clonogenic formation assay

Based on our previous studies, different numbers of HUVEC and L02 cells were plated in 6-well plates, and then were exposed to 0, 2, 4, 8Gy irradiation as indicated. Then cells were incubated until they formed colonies with at least 50 cells each. The colonies were rinsed with PBS and stained with methanol/crystal violet dye. Colonies with >50 cells were scored as a surviving colony.

### Apoptosis assay

At 24h post-irradiation (8Gy) with and without HKST treatment, apoptosis of HUVEC and RAW264.7 cells were determined by double-staining with Annexin V-fluorescein isothiocyanate (Annexin V-FITC) and Propidium Iodide (PI) using a Apoptosis Detection Kit (Invitrogen, Carlsbad, California, USA) and analyzed by flow cytometry (Beckman Cytoflex) according to the manufacturer’ instructions [21].

### Immunofluorescence analysis

We used an immunofluorescence assay to detect γH2AX foci (DNA double strand break marker) and the subcellular location of NF-kB p65 subunit. Briefly, RAW264.7 cells were seeded on 22×22mm^2^ cover glasses in 6-well plates at the concentration of 2×10^5^ per well. After HKST treatment, cells were exposed to 3Gy irradiation, and 0, 0.5, 8, 24h later, cells were fixed in 4% paraformaldehyde for 20min and permeabilized in 0.5% Triton X-100 for 10min. After blocked in serum, cells were stained with γH2AX and NF-kB p65 primary antibody (Santa Cruz, US; 1:200) and then with the secondary antibody (1:1000). Cellular images were obtained using an Olympus BX60 fluorescent microscope (Olympus America Inc., Center Valley, PA, USA) equipped with a Retiga 2000R digital camera (Q Imaging Inc., Surrey, BC, Canada). Image Pro Plus (Media Cybernetics, Silver Springs, MD) were used to count the γH2AX foci per cell according to our previous studies [22], and at least 100 cells per group were counted.

#### Animal survival assay

Mice pretreated with PBS or HKST were exposed to 7Gy total body irradiation at a dose rate of 1Gy/min. Then mice were returned to animal house, in which they were housed in the same facility, and the survival rate was recorded for 30 days in the same condition.

### Flow cytometry analysis of bone marrow cells and splenocytes

To measure the number of nucleated cells and CD34+ cells, cells isolated from each femur per mice were collected and the red blood cells were dissociated and removed as previous described [23]. The total number of nucleated cells was calculated with a flow cytometry. For CD34+ haematopoietic stem cells (HSC) detection, nucleated cells from bone marrow were stained with CD34-FITC and B220-PE antibodies and subjected to flow cytometry analysis. To determine the ratio of CD4+ and CD8+ cells, splenocytes were stained with CD4-FITC and CD8-PE and analyzed with flow cytometry.

### Tissues isolation and Haematoxilin-Eosin (HE) staining

On day 1, 3, 7 post total body irradiation, spleen, femur and testis were isolated, fixed and subjected to sectioning. A HE staining method was applied to detect tissue damages. For TLR2−/− and TLR4−/− mice, tissues samples were isolated on day 3 post total body irradiation and the same staining method was applied [23].

### Enzyme-linked immunosorbent assay (ELISA)

As a result of exposure to IR, multiple inflammatory cytokines are upregulated, such as tumor-necrosis factor α (TNF-α), interleukin-6 (IL-6) and IL-1β etc. It was known that IR also causes an increase of T helper 2 (Th2) response, while Th1 response is inhibited. To explore the influence of HKST on radiation-induced inflammation, we examined the level of cytokines in blood serum. On the third day post-irradiation, blood serum was isolated and subjected to an ELISA assay according to the manufacturer’s instructions (Westang Tech., Shanghai, China).

### Antibodies and western blotting analysis

At 0, 0.5, 8h post-irradiation, proteins from HUVEC cells were obtained by using ProtecJETTM Mammalian Cell Lysis Reagent (Fermentas, Vilnius, Baltic, Lithuania) according to manufacturer’s protocol, and then analyzed by western blotting to detect γH2AX (Cell Signaling Tech., 1:1000), TRIF (Proteintech, Wuhan, China; 1:1000), MyD88 (Abcam, US; 1:1000), p-Erk, p-Jnk, p38 (Sampler kit from Cell signaling technology, US; 1:1000), and GAPDH (Proteintech, Wuhan, China; 1:1000). The secondary antibody (1:1000) was also purchased from Cell Signaling Technology.

### Statistical analysis

Data were shown as means ± standard error of mean (SEM) for each experiment. The number of samples is indicated in the description of each experiment. We used an analysis of variance (ANOVA) followed by a Student-Newman-Keuls post hoc test for statistical analysis. Experiments for quantification were conducted in a blinded fashion and all the experiments were repeated for at least 3 independent times.

## Abbreviations

HKST: heat-killed salmonella typhimurium
TLR: toll like receptor
IR: ionizing radiation
LPS: lipopolysaccharide
DSB: double-strand breaks
HSC: hematopoietic stem cells
